# Sustainable Recycling of Mushroom Residue as an Effective Substitute for Cotton Hull Waste in *Volvariella volvacea* Cultivation: Evidence from Physicochemical and Microbiome Analyses

**DOI:** 10.3390/microorganisms13102372

**Published:** 2025-10-15

**Authors:** Pattana Kakumyan, Lin Yang, Shunjie Liu, Kritsakorn Saninjuk, Qin Dong, Xueyu Pan, Changxia Yu, Yan Zhao

**Affiliations:** 1 Institute of Edible Fungi, Shanghai Academy of Agricultural Sciences, Shanghai 201403, China; pattana.kak@mfu.ac.th (P.K.); ylin_jade@163.com (L.Y.); jasonliu86@foxmail.com (S.L.); maomao88719@163.com (Q.D.); panxueyu93@163.com (X.P.); ycx41529@163.com (C.Y.); 2 School of Science, Mae Fah Luang University, Chiang Rai 57100, Thailand; kritsakorn.san@mfu.ac.th; 3 Microbial Products and Innovations Research Group, Mae Fah Luang University, Chiang Rai 57100, Thailand

**Keywords:** *Volvariella volvacea*, mushroom residue of *Lyophyllum decastes*, composting, microbial diversity, physicochemical attributes

## Abstract

Mushroom residue (MR) is extensively produced during the industrialized cultivation of mushrooms, and its utilization is environmentally sustainable. Cotton hull waste (CW) serves as a common raw material for the cultivation of *Volvariella volvacea* in China. This study compared MR- and CW-based cultivation formulas with respect to their physicochemical characteristics, bacterial communities, and functional dynamics during substrate fermentation (composting). Xylanase production was greater in the MR formula than in the CW formula. Conversely, cellulase (CMCase) was generated at higher levels in the CW formula compared to the MR formula. Interestingly, the biological efficiency of MR was found to be comparable to that of CW, but the cost of MR was much lower. The dynamics of bacterial communities and their associated metabolic functions during substrate fermentation were monitored using 16S rRNA metagenomics techniques. Significant alterations in bacterial community structure were observed within both formulas throughout the preparation phase. Indicator species analysis revealed distinct patterns of bacterial diversity development between MR- and CW-based composts during fermentation. Metabolic function analysis indicated that carbohydrate and amino acid metabolism remained relatively active throughout this process. These results suggest that the MR formula is equally effective as conventional CW compost for supporting *V. volvacea* cultivation, while also offering a lower raw material cost.

## 1. Introduction

*Volvariella volvacea* (Bull. ex Fr.) Singer, also known as straw mushroom, is one of the major commercial edible mushrooms cultivated in China. This mushroom is popular because it contains high levels of nutrients with abundant proteins and produces a pleasant flavor and taste. *V. volvacea* grows on various substrates, and straw and cotton hull waste (CW) are usually used as substrates for commercial production [1]. Substrate fermentation is widely used, as it can increase mushroom production. The maturity of fermented substrates is determined by many factors, including the substrate’s temperature, humidity, and nutrients such as the C/N ratio [2].

Mushroom residue (MR) is a substrate colonized by fungal mycelia after mushroom harvesting. Its composition varies depending on the mushroom species cultivated, typically containing straw, sawdust, and gypsum, as well as nutrients such as nitrate and phosphate [3]. The mushroom industry produces approximately 64 million tons of MR globally, with each kilogram of fresh mushrooms producing approximately 3 to 5 kg of MR [4]. In 2010, China produced more than 13.2 million tons of MR, which led to a significant waste disposal challenge [5]. MR can be reused as an alternative substrate for new mushroom cultivation [6]. For example, the MR of *Lentinula edodes* [7], *Hypsizygus marmoreus* [8], and *Pleurotus ostreatus* [9] can be reused as substrates for the cultivation of *Pleurotus* species. Therefore, the recycling of MR by introducing it into substrates for the production of other mushrooms is an environmentally friendly way to recycle mushroom residue waste.

During the fermentation process, different raw materials can lead to variations in microbial communities and shifts in dominant species and functional groups [10]. Meanwhile, changes in microbial communities influence the degradation of organic matter, which further affects the maturation of the fermentation substrate. Thus, microbial communities are very important in the composting process [11]. The role of microbial communities during substrate fermentation in mushroom growth has been extensively studied, including species such as *Agaricus bisporus* [12,13] and *Pleurotus ostreatus* [10,14]. The analysis of dynamic changes and metabolic functions within the microbiota during the fermentation process is important for elucidating relationships among substrate physicochemical properties, microbial communities, and microbial metabolism [10,15]. In recent years, culture-independent molecular analysis, metagenomic sequencing-generated functional genomic datasets, and metabolomic approaches have been used to identify the metabolic functions of consortia in cultivation systems [10,14,16]. Meanwhile, these results provide an overview of the functions of environmental microbial communities in substrate preparation for mushroom cultivation. Nevertheless, although the relationships among the physicochemical properties, microbial communities, and metabolic functions have been widely reported in previous studies, their relationships during the composting process of *V. volvacea* strains are poorly defined. Therefore, from the perspective of microorganisms, studying the changes in microbial diversity, microbial functions, and the relationships between them and the physicochemical properties of the substrate is of great significance for understanding the key factors that affect *V. volvacea* cultivation and for subsequently promoting a high-efficiency process for *V. volvacea* mushroom production.

Determining the changes in physicochemical parameters, lignocellulose-degrading enzyme activities, bacterial communities, and bacterial metabolic functions during the composting process can both clarify how mushroom residue affects *V. volvacea* cultivation and its underlying mechanisms and provide a theoretical basis for utilizing other spent mushroom substrates. Therefore, the objective of this study is to investigate the variations in the physicochemical properties and lignocellulose-degrading enzyme activities with respect to bacterial communities and metabolic functions in different composting compositions and stages of substrate preparation for *V. volvacea* cultivation. These relationships with respect to biological efficiency are also compared. It is expected that there will be significant differences in the physicochemical properties, bacterial communities, and bacterial metabolic functions between the CW and MR formulas.

## 2. Materials and Methods

### 2.1. Materials and Mushroom Strains

Cotton hull waste (CW) was obtained from an agricultural company in Zhenjiang, Jiangsu Province, China, and the price of CW was 1200 CNY/t. MR of *Lyophyllum decastes* was collected from Pudong New Area, Shanghai, China, and used along with rice straw as a supplemented substrate for the cultivation of *V. volvacea*. Strain V23 was described by Wang et al. [8]. The price of MR was 75 CNY/t.

### 2.2. Composting Process and Sampling

There were two groups of substrates in this research. The first group, without the addition of MR (97% CW, 3% lime), was regarded as the control treatment for comparison; the second group was regarded as the treatment and included the addition of MR for composting with rice straw (50% MR, 47% rice straw, 3% lime). The fresh samples were soaked with lime water and maintained at approximately 70% water content. The samples were divided into six parallel trials, with 8 kg/basket (490 mm × 370 mm × 100 mm) for the CW formula and 15 kg/basket (580 mm × 390 mm × 180 mm) for the MR formula. Samples from three phases were collected for sequencing using the multipoint sampling method (see Supplementary Materials). The first sample was collected after prewetting the substrate material with lime water and adjusting the moisture of the raw materials (BH, P0). The mixture was then transported into a sealed sterilization room. The second sample (PI) was collected after 1 day of fermentation at room temperature (1st fermentation). The door of the fermentation room was then opened for ventilation and resealed. High-temperature steam was passed through the material until the temperature reached 60 °C, after which the temperature was maintained for 10 h (2nd fermentation). After the 2nd fermentation (the end of phase II), a third sample (PII) was collected. Each collected sample was used for the determination of physicochemical properties and 16S rDNA sequencing.

### 2.3. Determination of Mushroom Yields

Mature composts of the CW substrate and MR with rice straw substrate were used for *V. volvacea* cultivation. The mature composts were inoculated with 1% spawn culture. The relative humidity of the air was controlled at approximately 90% during mushroom production, and the temperature of the mushroom house was maintained at 32 °C throughout the cultivation process. The harvest period started from day 14 after the spawn-running stage and continued for a total of 7 days. Mushroom fruiting bodies with an egg shape were harvested manually and weighed. The total mushroom yields (g/kg substrate) and biological efficiency values (%BE) were calculated according to Song et al. [17].

### 2.4. Assays of Physicochemical Properties

Samples from six phases (P0, PI, PII, Fi, PF, and Ha) were collected for physicochemical analysis. The electrical conductivity (EC), pH, ash content, and water content were determined according to Song et al. [16]. The pH and total nitrogen (TN) content were determined using a digital pH meter (LAQUA twin-pH-22 pH meter, Horiba Corporation, Kyoto, Japan) and an automatic Kjeldahl nitrogen analyzer (UDK169, VELP, Milan, Italy), respectively. The ash content and total carbon (TC) content were measured by determining the loss on ignition at 150 °C to a constant weight in a muffle furnace and K_2_Cr_2_O_3_ oxidation, respectively. The TC and TN values were used to calculate the C/N ratios. Cellulose, hemicellulose, and lignin were measured, and carboxymethyl cellulase (CMCase), xylanase, and laccase activities were also analyzed. The contents of lignin, hemicellulose, and cellulose were tested according to Van Soest’s method [18]. The supernatants used to determine CMCase, xylanase, and laccase activities were collected according to Kong et al. [10]. Briefly, the compost sample was mixed with 0.85% NaCl and then filtered and centrifuged to obtain the supernatant. CMCase activity was tested using CMC sodium salt as a substrate, and the reducing sugars released by cellulase were measured using the anthrone–H_2_SO_4_ method. Xylanase activity was obtained by adding the supernatant to xylan and then measuring the OD value of the product from the enzyme reaction at 540 nm via the dinitrosalicylic acid method. Laccase activity was assayed by the oxidation of ABTS, which was monitored by determining the increase in the A420 value (ABTS, ε420 = 36,000 M^−1^ cm^−1^).

### 2.5. Bioinformatics Analyses of Bacterial Microbiota Diversity

The total genomic DNA of the compost samples was extracted and quantified according to the report by Gu et al. [19]. For extraction of total genomic DNA from compost samples, the samples were extracted using cetyltrimethylammonium bromide (CTAB) following the manufacturer’s instructions. The 16S rRNA gene V4 region was amplified with barcoded primers 515F (5′-GTGYCAGCMGCCGCGGTAA-3′) and 806R (5′-GGACTACNVGGGTWTCTAAT-3′). After purification, all the PCR products were sequenced on the IonS5 ^TM^ XL platform (Thermo Fisher Scientific, Waltham, MA, USA).

The raw sequencing data were processed with the QIIME2 (v2024.5.0) pipeline. First, the adapter and primer sequences were trimmed using the Cutadapt plugin, and then high-quality amplicon sequence variants (ASVs) were generated using the DADA2 plugin for downstream analysis. Data visualization was carried out using the *ggplot* and *pheatmap* packages in R software (version 2.15.3).

The statistical comparisons of alpha diversity, beta diversity, and linear discriminant analysis effect size (LEfSe) were calculated and visualized using the *phyloseq* package in R software [20]. The Kruskal–Wallis sum-rank test was also used to detect significant bacterial biomarkers across sample groups. The Spearman correlation coefficient between microbial species and physicochemical properties was calculated in R software.

### 2.6. Statistical Analysis

All compost samples were analyzed five times. For the other parameters (water content, pH, EC, ash, TC, and TN), 3 replicates were used, and the data were processed by GraphPad Prism 7. SPSS 25 (SPSS for Windows, USA) was used to analyze the statistical significance of the properties in the different treatments. Significant between-group analysis was conducted using Duncan’s multiple range test. The results are shown as the means ± standard deviations.

## 3. Results and Discussion

### 3.1. Physicochemical Changes During Composting

**Figure 1:**
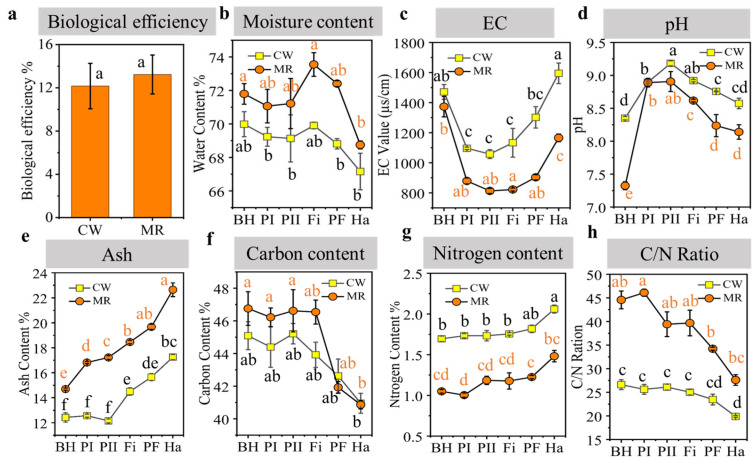
Physicochemical properties during the composting and mushroom growth stages: (**a**) biological efficiency, (**b**) moisture content, (**c**) EC, (**d**) pH, (**e**) ash, (**f**) carbon content, (**g**) nitrogen content, and (**h**) C/N ratio. Different lowercase letters indicate significant differences among treatments (*p* < 0.05).

Figure 1 illustrates the variations in the physicochemical characteristics of compost and mushroom production. As depicted in Figure 1a, significance analysis showed that there was no difference in the biological efficiency of MR and CW, which indicates the feasibility of MR as a culture medium for *V. volvacea*. Meanwhile, the price of MR (75 CNY/t) is much lower than that of CW (1200 CNY/t), so under the same biological conversion efficiency, using MR as the substrate yields greater economic benefits than using CW. During the composting process, the moisture content of the substrate did not change significantly. Following primordium formation, water was sprayed to maintain humidity, thereby promoting *V*. *volvacea* growth. Consequently, the water content significantly increased during the Fi phase before decreasing during fructification (Figure 1b), which enhanced the porosity of the compost feedstock and subsequently increased oxygen levels within the heap [10]. The EC value reflected the total ion concentration in different samples (Figure 1c). Both CW and MR exhibited a decrease in EC values during composting, followed by an increase during the spawn-running and fructification phases. Furthermore, it was observed that the EC values for CW were higher than those reported by Zhou et al. [21], suggesting that reductions in EC values within compost may be attributed to transformations involving NH_4_^+^ and NO_3_^−^ ions; conversely, prolonged increases may result from mineral salt release due to organic matter degradation [22]. Elevated concentrations of soluble salts such as calcium nitrate and calcium chloride can negatively impact mushroom productivity [23]. Therefore, from this perspective, MR is deemed more suitable for mushroom cultivation. The pH increased continuously during the composting phase, from 8.35 to 9.18 in the CW formula and from 7.32 to 8.91 in the MR formula (Figure 1d). During spawn running and fructification, there was a persistent pH decrease (from 9.18 to 8.57 in the CW formula and from 8.91 to 8.14 in the MR formula). The microbial decomposition of organic matter during composting can lead to the generation of organic acids, resulting in a reduction in pH [10]. Therefore, in this study, during the high-temperature stage of the fermentation process, the organic matter was consumed, generating organic acids, which led to a decrease in pH value after the fermentation process. The ash content in the substrate, shown in Figure 1e, increased from the BH to the Ha phases and was greater in the MR formula than in the CW formula.

The carbon content of both formulas decreased during spawn running and fructification (Figure 1f), whereas the nitrogen content increased throughout the period (Figure 1g). The C/N ratio (Figure 1h) of the MR formula was greater than that of the CW formula, and both decreased as the cultivation process progressed. This may be attributed to the higher consumption rate of carbon compared to nitrogen [10]. As illustrated in Figure 1h, the observed C/N ratio is suitable for the growth of *V*. *volvacea,* as C/N ratios between 20 and 30 are considered optimal for vegetative growth and spawning on cotton hull waste substrates [24]. Furthermore, the C/N ratio (27–45) in the MR formula also supports *V. volvacea* growth since its biological efficiency was not lower than that of CW (Figure 1a).

### 3.2. Changes in Lignocelluloses and Related Degrading Enzymes

**Figure 2:**
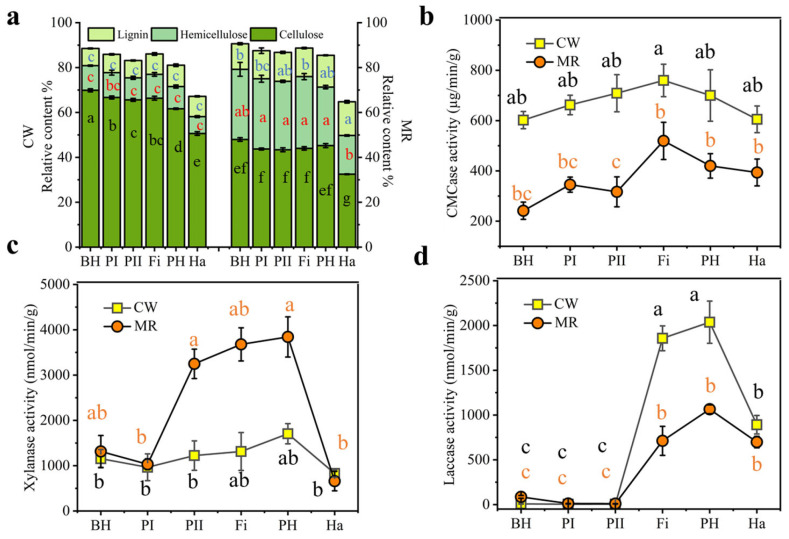
Relative lignocellulose content (**a**) and lignocellulose-degrading enzyme activities during the composting and mushroom growth stages with different substrate formulas: (**b**) cellulase (CMCase) activity, (**c**) xylanase activity, and (**d**) laccase activity. Different lowercase letters indicate significant differences among treatments (*p* < 0.05).

The contents of lignin, hemicellulose, and cellulose in both the CW and MR samples were analyzed and are presented in Figure 2a. The content of cellulose in both formulas exceeded 40%, followed by hemicellulose, with lignin being the least abundant. The cellulose content of CW was greater than that of MR, whereas the hemicellulose and lignin contents of CW were lower than those of MR.

Carboxymethyl cellulase (CMCase) plays a key role in cellulose degradation [25]. The CMCase activity in the CW formula was greater than that in the MR formula, and both reached their highest values in the Fi phase and then gradually decreased (Figure 2b). Xylanase is essential for hemicellulose degradation [25]. As shown in Figure 2c, xylanase activity was higher in MR than in CW; the MR formula reached its maximum activity between the PII and pH phases, which subsequently decreased during the Ha phase, while the CW formula maintained constant activity throughout the process. Laccase functions as a phenol oxidase and is part of the lignin-degrading enzyme system [10]. The laccase activity of CW was greater than that of MR, and its content was very low during the composting phase and then increased after the primordium formed, peaking during the Fi and PH phases and then decreasing. The variations in laccase, xylanase, and CMCase activities observed in both formulas were consistent with the respective quantities of lignin, hemicellulose, and cellulose present. The early arrival of the cellulase peak indicated that cellulose was the initial substrate to undergo degradation. The lack of laccase activity during the composting phase indicated that *V. volvacea*, rather than bacteria, was primarily responsible for lignin production.

### 3.3. Evolution of the Microbial Community During Composting

The V4 region of the 16S rRNA gene was used for amplicon sequencing to identify and characterize bacterial diversity during the composting process. Using the amplicon sequence variant (ASV) approach, we analyzed the microbial community dynamics across different composting stages. Consistent with the observed rarefaction curve asymptotes, the ASV frequencies reached a plateau at higher sequencing depths, indicating satisfactory sampling saturation (Figure S1). A total of 1267 ASVs were identified across the 30 compost samples.

**Figure 3:**
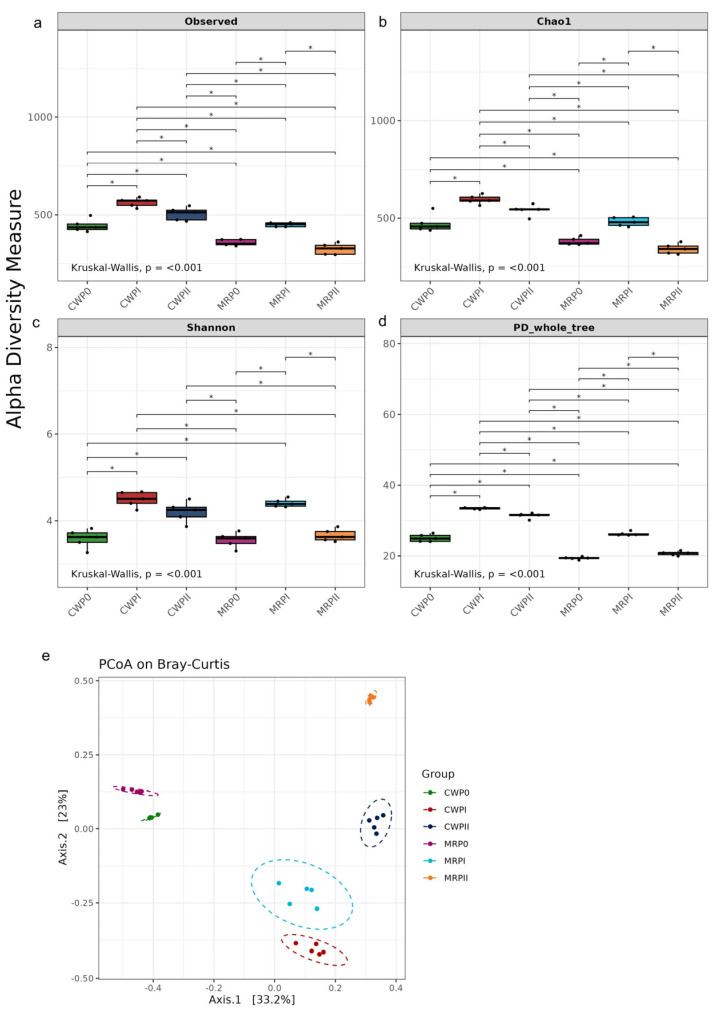
Alpha and beta diversity of the bacterial community in the CW and MR samples. The alpha diversity indices, including the observed ASVs (**a**), Chao1 (**b**), Shannon (**c**), and Faith’s phylogenetic diversity (PD whole tree) (**d**), across different composting phases for the CW and MR formulas. Principal coordinate analysis (PCoA) based on Bray–Curtis dissimilarity revealed clustering patterns of bacterial communities across treatments (**e**). PERMANOVA was used to analyze statistical differences in community composition for all pairwise comparisons, and the results are indicated with asterisks (*p* < 0.05).

Alpha diversity analysis demonstrated significant variations in both microbial richness and diversity when comparing the CW and MR formulas. Compared with the MR formula, the CW formula resulted in a significantly greater number of observed species (Figure 3a). The Chao1 index, which represents species richness, consistently presented higher values in the CW formula throughout the composting process, whereas the MR formula reached its peak richness in PII (Figure 3a). This trend suggests that the CW formula maintained a richer microbial community over time. The Shannon index, which measures species diversity, showed similar trends for both formulas, with diversity peaking during the PI phase, indicating that microbial diversity was highest in this composting stage. The phylogenetic diversity (PD whole tree) followed a similar trend, with the CW compost maintaining greater phylogenetic diversity than the MR compost throughout the composting process.

Beta diversity analysis via Bray–Curtis principal coordinate analysis (PCoA) revealed distinct microbial community structures among the different composting formulas and phases. The clustering pattern suggests that the composting phase strongly influenced community composition, with early (P0), mid (PI), and late (PII) phases forming distinct clusters, particularly for the MR formula. This difference highlights the impact of substrate composition and environmental factors on microbial succession during composting. Notably, MRPII showed the greatest divergence, with the highest F-values, such as in comparisons with CWP0 (F = 87.199, R^2^ = 0.9159, *p* = 0.001) and MRP0 (F = 84.724, R^2^ = 0.9137, *p* = 0.001). Comparisons involving CWPI also revealed substantial differences, such as CWPI vs. MRPII (F = 67.549, R^2^ = 0.8941, *p* = 0.013). These results align with the NMDS clustering patterns and indicate that the bacterial communities in CWPI and MRPII were highly distinct. These findings highlight that the type of substrate and fermentation stage significantly affected the bacterial community structure and diversity. For example, the CW formula fostered greater microbial diversity, whereas the MR formula presented unique community dynamics.

**Figure 4:**
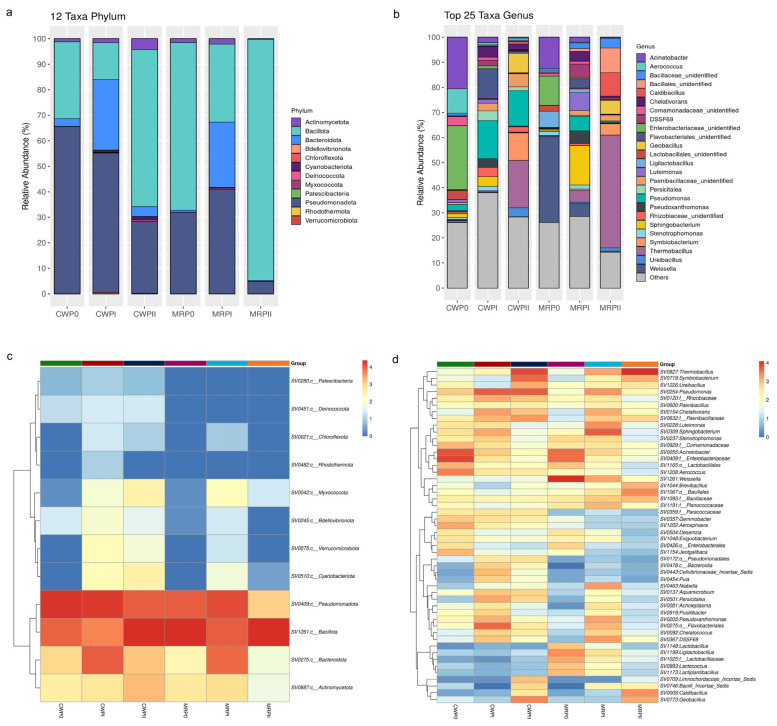
The composition of the bacterial community at the phylum and genus levels across different composting phases and substrate formulas. Relative abundance of the 12 bacterial phyla in the CW (CWP0, CWPI, and CWPII) and MR (MRP0, MRPI, and MRPII) samples during the P0, PI, and PII phases of composting (**a**). Relative abundance of the top 25 bacterial genera in the CW and MR formulas during the P0, PI, and PII phases (**b**). Heatmap of phylum-level relative abundance in the CW and MR formulas during the P0, PI, and PII phases (**c**). Heatmap of genus-level relative abundance in the CW and MR formulas during the P0, PI, and PII phases (**d**).

The dynamics of the top 12 predominant bacterial phyla during the composting phase for each substrate formula in this study revealed that Bacillota, Pseudomonadota, Bacteroidota, and Actinomycetota were common across all composting processes, as illustrated in the phylum-level bar plot (Figure 4a,c). Key phyla, including Bacillota, Pseudomonadota, and Bacteroidota, exhibited dynamic shifts based on the type of substrate and composting phase. Pseudomonadota was the predominant phylum in the CW formula during the P0 (65.39 ± 4.32%) and PI (54.40 ± 2.61%) phases, but Bacillota became the dominant phylum during PII (61.650 ± 2.46%). In contrast, the MR formula exhibited a different pattern, with Bacillota predominating in both the P0 (65.60 ± 6.71%) and PII (94.54 ± 1.48%) phases. During the PI phase, Bacillota, Pseudomonadota, and Bacteroidota were present in the MR formula at comparable levels, with relative abundances of 30.60 ± 7.97%, 40.87 ± 6.59%, and 25.50 ± 4.35%, respectively. These findings indicate that the MR substrate with rice straw promoted the growth of Bacillota throughout the fermentation process compared with the CW substrate. The enhanced survival capacity of Bacillota in the MR formula is attributable to their ability to produce heat-resistant endospores, as reported by Yang et al. [26] and Zhong et al. [27]. Moreover, the relative abundance of Bacteroidota in both formulas temporarily increased during the PI phase before gradually declining in PII, which contrasts with the findings of Guo et al. [15].

Figure 4b,d illustrate the genus-level dynamics within bacterial communities throughout the composting process. In the initial composting phase (P0), the bacterial community of the CW formula was predominantly composed of Pseudomonadota (65.39 ± 4.32%), with *Acinetobacter* (20.54 ± 3.18%) representing the most abundant genus. In contrast, the MR formula exhibited a greater abundance of the phylum Bacillota (65.60 ± 6.71%), which was predominantly represented by the genus *Weissella* (34.15 ± 3.00%), than the phylum Pseudomonadota (32.17 ± 7.14%), which included *Acinetobacter* (12.60 ± 2.14%).

These findings align with those of previous studies by Martins et al., which reported a high abundance of *Acinetobacter* associated with lignocellulosic degradation in waste composting environments [28]. In support of this observation, cellulase and xylanase activities were consistently observed throughout the mushroom production cycle (Figure 2b,c), further supporting the critical role of these microbial communities in substrate decomposition and nutrient cycling.

Compared to the initial phase (P0) and the thermophilic phase (PII) of composting, the bacterial community structure of the genera in phase I (PI) showed little difference between the CW and MR formulas. At the phylum level, the bacterial communities could be categorized into three predominant groups: Pseudomonadota, Bacillota, and Bacteroidota (Figure 4a,c). The most abundant genus in the CW formula during PI was *Pseudomonas* (15.01 ± 3.86%). This genus presented higher relative abundances in the CW formula than in the MR formula. In contrast, the most abundant genera in the MR formula during PI were *Sphingobacterium* (15.64 ± 3.00%), *Pseudoxanthomonas* (4.92 ± 1.06%), and *Chelativorans* (3.80 ± 0.64%) (Figure 4b,d). Previous studies have shown that *Bacteroidota* and *Pseudomonadota* play significant roles in the decomposition of organic matter during the cooling and maturity stages of composting [26]. Similarly, Pseudomonadota and Bacillota presented the highest diversity during phase I of sugarcane straw-based composting, with *Pseudoxanthomonas*, *Pseudomonas*, and *Bacillus* being predominant [29]. These genera are notable for their thermotolerance and roles in carbon cycling and nitrogen conversion [30]. Multiple studies have demonstrated that Pseudomonadota, Bacteroidota, and Actinomycetota play crucial roles in carbon and nitrogen transformation and cellulose and lignocellulose degradation [27,31].

The difference in community structure may result from the different substrate materials used in the CW and MR formulas. This finding contrasts with the findings of Vieira et al. [29], who reported reductions in the abundance of Actinomycetota, Bacteroidota, and Pseudomonadota during phase I of the composting of sugarcane straw supplemented with wheat bran; these abundances progressively increased during phase II of composting. Additionally, the genus *Bacillus*, identified in this study, is associated with metabolic pathways, including butanoate metabolism and the fermentation of acetyl-CoA to butyrate [32].

In phase II (PII) of substrate fermentation, the dominant bacterial genera in the CW formula were *Thermobacillus* (19.00 ± 4.56%), *Symbiobacterium* (11.02 ± 6.68%), and *Pseudomonas* (13.97 ± 5.01%). The most abundant genera in the MR formula were *Thermobacillus* (45.26 ± 9.59%), *Caldibacillus* (9.35 ± 2.62%), and *Symbiobacterium* (4.53 ± 0.92%) (Figure 4b,d). *Caldibacillus* is recognized for its production of heat-resistant mannanase, which, in conjunction with cellulase and xylanase, effectively degrades plant cell walls and promotes the release of nutrients. *Sphingobacterium* and *Pseudomonas* are bacteria that are associated with nitrogen conversion processes [33]. The activities of nitrogen-transforming bacteria may result in elevated nitrogen levels during composting. The abundance of *Thermobacillus*, a genus known for its ability to produce xylanase [34], increased during PII of composting for both formulas. The relative abundance of this genus was significantly higher in the MR formula (45.26 ± 9.59%) than in the CW formula (19.00 ± 4.56%). These results revealed that the addition of mushroom residue and rice straw facilitated the growth of Bacillota, particularly *Thermobacillus*, in PII, resulting in increased lignocellulose degradation. Interestingly, Actinomycetota, which was reported as being essential for lignocellulose degradation, was detected in all phases of composting; however, it was not the dominant phylum in this study [35]. Guo et al. identified Bacillota, Actinomycetota, Pseudomonadota, Chloroflexi, and Bacteroidota as the main phyla during composting [15].

The diversity of the bacterial community reflected its adaptation to the composting environment, with initially low-abundance bacteria proliferating after composting started [29]. A shift in the bacterial community as the composting process progressed was observed. Throughout several phases of the composting process, there was considerable enrichment at the phylum, class, and genus levels, along with certain bacterial groupings. Our findings revealed that Pseudomonadota were predominantly enriched in PI and PII in the CW formula. The higher linear discriminant analysis (LDA) score for the CW formula was attributed to Bacillota, not Pseudomonadota, which presented a better score for the MR formula than the CW formula (Figure S2a,b). The LDA revealed significant differences in bacterial taxa abundance between the two substrate formulas, indicating that mushroom residue with rice straw promotes the proliferation of Bacillota. These findings suggest that the substrate composition significantly influences microbial community development during composting.

### 3.4. Analysis of Microbial Community Functions

**Figure 5:**
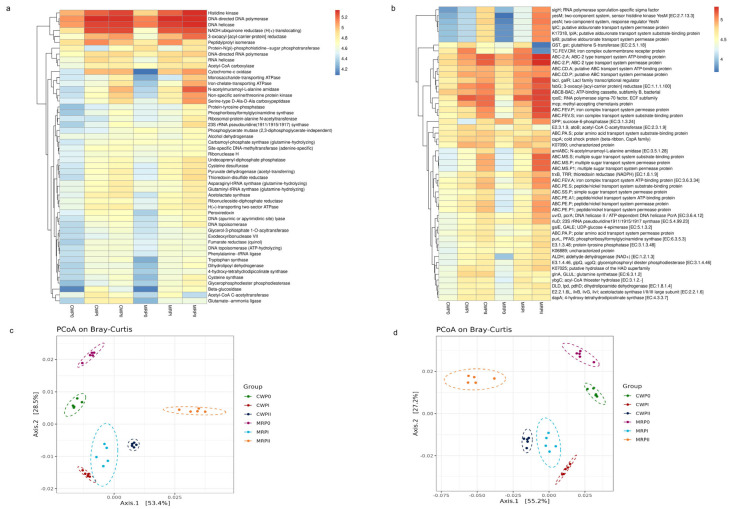
Metabolic functional prediction profiles during the composting phase (P0, PI, PII) for both the CW and MR formulas, annotated by PICRUSt2. Heatmap showing the relative abundance of functional profiles with the EC database (**a**). Heatmap displaying the relative abundance of functional profiles with KEGG level 3 (**b**). Principal coordinate analysis (PCoA) based on the Bray–Curtis dissimilarity of the functional gene profiles of EC and KEGG (**c**,**d**). The ellipses represent 95% confidence intervals.

The predicted bacterial functions related to microorganisms were classified and summarized, as shown in Figure 5a,b. At KEGG level 1, the dominant predicted pathways were associated primarily with metabolism, environmental information processing, genetic information processing, and cellular processes. Among these pathways, metabolic functions accounted for the greatest proportion of all composting phases and substrate formulas, suggesting the importance of microbial communities in organic matter decomposition and nutrient cycling. Carbohydrate metabolism, amino acid metabolism, and energy metabolism were the most abundant metabolic pathways observed in all the samples (Figure S3).

KEGG level 3 metabolic pathway analysis revealed distinct functional profiles throughout the composting process between the CW and MR formulas. The heatmap highlights the relative abundance of key metabolic pathways, including amino acid metabolism, carbohydrate metabolism, lipid metabolism, cofactor and vitamin metabolism, nucleotide metabolism, and xenobiotic biodegradation and metabolism. Notably, the relative abundance of the carbohydrate and amino acid metabolism pathways consistently increased from the initial composting phase (P0) to the intermediate (PI) and maturation (PII) stages, suggesting an active role in substrate transformation.

Among the various metabolic pathways detected, amino acid and carbohydrate metabolism were the primary metabolic pathways observed and were consistently present throughout all composting phases in both formulas. During the composting phases (PI and PII), the relative abundance of the carbohydrate and amino acid metabolism pathways was greater than that in the initial composting phase (P0). This suggests that microbial activity increased throughout the composting process compared to that in the raw materials (Figure 5a). A comparison of the physicochemical properties of the two formulas revealed that the CW formula exhibited more total nitrogen (TN) than the MR formula. This may explain why amino acid metabolism was more prevalent in the CW formula. This finding aligns with previous studies on composting processes based on substrates containing rice straw or maize straw [21,36,37]. Amino acid metabolism affects amino acid production and the synthesis of humic substances [21,38].

As reported, carbohydrate metabolism and amino acid metabolism were the predominant metabolic pathways in various composting processes, including the composting of whole green soybean hulls [39] and swine manure composting [40]. Carbohydrate metabolism plays an important role in hemicellulose degradation by producing various compounds from the breakdown of hemicellulose and cellulose [41]. Additionally, elevated TN content has been shown to promote the growth and reproduction of composting microbes in peach sawdust-based composting [15]. The significant functional differences between the two formulas in different composting stages were analyzed (*p* < 0.05).

Pathway enrichment analysis was carried out with the KEGG IDs of the differentially abundant metabolites to explore the origin of these metabolites and assess how substrate composition affects microbial functions in each phase of the composting system. The results of metabolic pathway enrichment (Figure 5b) revealed significant alterations in the microbial metabolic pathways related to protein digestion and absorption (digestive system), membrane transport (ABC transporters), and amino acid biosynthesis. ABC transporters, which facilitate substrate transport across cell membranes [42], showed higher activity in the MR formula than in the CW formula. Notably, genes associated with the ABC transporter system, such as ABC-2 and ABC.CD, ABC.FEV (iron complex transport system), ABC.MS (multiple sugars), and ABC.PE (peptide/nickel), were more abundant in the PI and PII stages than before composting (P0). This increased ABC transporter activity indicates progressively complex substance transport dynamics within the composting system. Different raw materials contain different compositions of lignin, cellulose, hemicellulose, protein, and starch. Proteins involved in lipid biosynthesis were enriched during the composting phase, which was correlated with the increased abundance of Bacteroidota observed during PI and PII. This finding aligns with previous research indicating that Bacteroidota participates in the metabolism of linoleic acid during composting [43].

The beta diversity of microbial functional prediction profiles via principal coordinate analysis (PCoA), which is based on the Bray–Curtis dissimilarity of functional gene profiles for EC (Figure 5c) and KEGG (Figure 5d), revealed that the functional prediction profiles were grouped differently by composting phases and substrate formulas. The CW formula samples (CWP0, CWPI, and CWPII) were more closely grouped, which suggests that the microbial community structure was more stable and uniform. On the other hand, the MR formula samples (MRP0, MRPI, and MRPII) were more dispersed, which indicates that the microbial composition was more variable. The ellipses in Figure 5c,d indicate the differences between the CW and MR formulas. These findings support the idea that the composition of the substrate has a significant effect on the structure and function of microbes during composting. These results reveal that there is a strong connection between the functionality of microbes and the structure of their communities. This structure affects how well composting works and how nutrients are transformed.

### 3.5. Correlations Between Microbial Communities and Physiological Properties

**Figure 6:**
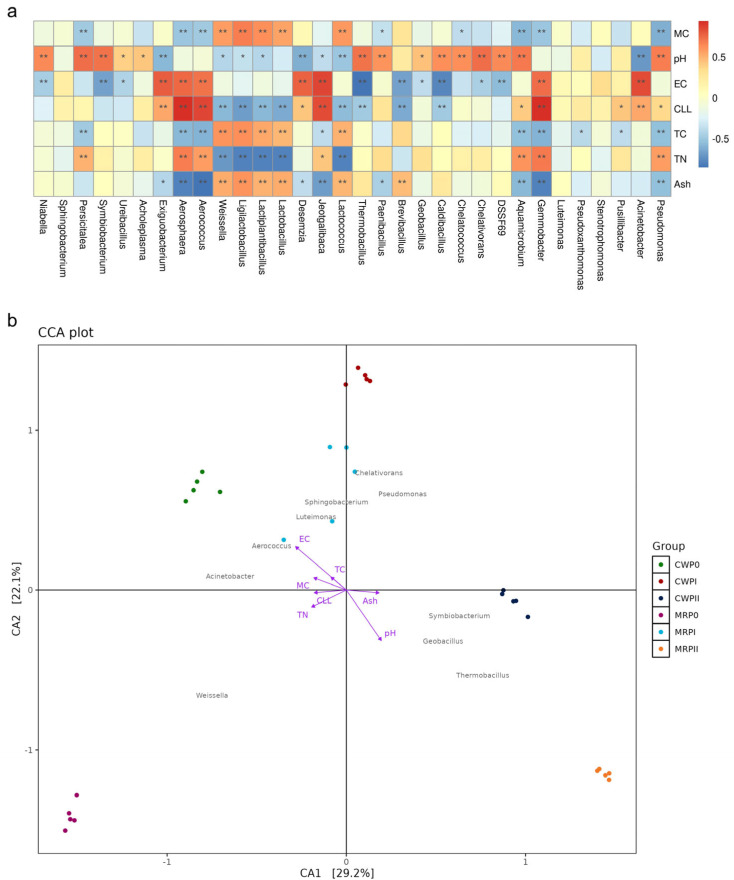
Correlations between microbial community composition and physiological properties during composting. (**a**) Heatmap showing Spearman’s correlation coefficients between the top 32 dominant bacterial taxa and environmental factors, including moisture content (MC), pH, electrical conductivity (EC), cellulose content (CLL), total carbon (TC), total nitrogen (TN), and ash content (Ash). Red indicates a strong positive correlation, and blue indicates a strong negative correlation. Asterisks (* and **) denote *p* values < 0.05 and *p* values < 0.01, respectively. (**b**) Canonical correspondence analysis (CCA) plot illustrating the relationships between microbial community composition and environmental parameters across different composting phases.

Exploring the relationships between microbial communities and environmental factors is highly important for improving the quality of composting products [2]. The top 32 dominant bacterial taxa were analyzed in relation to key environmental parameters, including EC, pH, C, N, and cellulose content (Figure 6a).

The bacterial taxa involved in lignocellulose degradation exhibited significant correlations with pH (Figure 6a). Members of the Bacillota phylum (e.g., *Acholeplasma*, *Ureibacillus*, and *Symbiobacterium*), the Bacteroidota phylum (e.g., *Persicitalea* and *Niabella*), and the Pseudomonadota phylum (e.g., *Pseudomonas*, *Aquamicrobium*, *DSSF69* (*Chitinophagaceae*), *Chelatococcus*, *Chelativorans*, *Caldibacillus*, *Geobacillus*, *Paenibacillus*, and *Thermobacillus*) demonstrated a significant positive relationship with pH. However, members of Bacillota (e.g., *Jeotgalibaca*, *Desemzia*, *Ligilactobacillus*, *Lactiplantibacillus*, *Weissella*, and *Exiguobacterium*) and Bacteroidota (e.g., *Acinetobacter* and *Lactococcus*) were negatively correlated with pH (Figure 6a).

The observed correlation patterns aligned with microbial succession during substrate composting. Pseudomonadota were very common in the first phase of composting (P0), when the pH was lowest. However, as composting continued (PI and PII), fewer compounds were found during PI, which was in line with the increase in pH. The abundance of *Thermobacillus* increased during PI, whereas the abundance of *Acinetobacter* decreased significantly.

The opposite trend was observed for EC. The abundances of *Acinetobacter*, *Gemmobacter*, *Aerococcus*, *Aerosphaeria*, and *Exiguobacterium* were positively correlated with water content. In contrast, the abundances of *DSSF69 (Chitinophagaceae)*, *Chelativorans*, *Caldibacillus*, *Geobacillus*, *Brevibacillus*, *Thermobacillus*, *Ureibacillus*, *Symbiobacterium*, and *Niabella* were significantly negatively correlated with water content. The abundances of *Pseudomonas*, *Acinetobacter*, *Pusillibacter*, *Gemmobacter*, and *Aquamicrobium* (classified under Pseudomonadota), along with *Jeotgalibaca*, *Desemzia*, *Aerosphaera*, *Exiguobacterium*, and *Aerococcus* (Bacillota), were positively correlated with the lignocellulosic content (CLL) (Figure 6a). These taxa were highly abundant in both composting formulas, suggesting their active role in lignocellulose degradation.

The canonical correspondence analysis (CCA) plot (Figure 6b) illustrates the relationships between microbial communities and environmental factors across different composting formulas and phases. The abundances of *Thermobacillus*, *Symbiobacterium*, and *Geobacillus* were positively correlated with pH and ash content, indicating that these thermophilic bacteria grow under conditions with increased alkalinity and ash accumulation, particularly in the PI and PII phases. This finding aligns with that of a previous study, which revealed that the abundance of *Thermobifida*, the dominant cellulose degrader, was positively correlated with the change in pH during composting [44]. *Thermobacillus* and *Symbiobacterium*, known for lignocellulose degradation, became dominant in later composting stages when the pH and ash content increased, reinforcing their thermophilic and cellulolytic roles, which aligns with previous research on organic waste composting [45].

In contrast, *Acinetobacter* and *Aerococcus* were negatively associated with these thermophilic bacteria but positively correlated with EC, revealing that these bacteria may play a role in the early composting phases when EC is higher. *Weissella*, which was dominant in the early composting stage (P0), was positioned away from TN, indicating that it decreased as the nitrogen content increased. On the other hand, lactic acid bacteria (LAB), including *Weissella*, *Ligilactobacillus*, *Lactiplantibacillus*, *Lactobacillus*, and *Lactococcus*, exhibited distinct correlations with environmental parameters during the composting process. This LAB group was significantly negatively associated with nitrogen content (TN) but positively associated with total carbon (TC) (Figure 6a). Weissella was most abundant in the MR formula in P0 but declined in PI and PII, which correlated with increasing nitrogen content during composting (Figure 4b). These findings revealed that nitrogen assimilation and amino acid metabolism were more active in PI during fermentation. Thus, these findings highlight the dynamic interactions between microbial communities across composting formulas and physicochemical conditions, providing insights into microbial contributions to composting efficiency.

## 4. Conclusions

In this study, variations in physicochemical properties led to the dominance of different genera at different composting stages. pH was the key factor at the beginning, whereas the C/N ratio became the main influencing factor during the later stages of composting. Slight differences in the biological efficiency of the CW- and MR-based formulas were observed, and the cost of the MR formula was lower. Changes in microbial community structure were related to different metabolic pathways, which were driven by different microorganisms at different stages between the two substrate formulas; however, only a few taxa accounted for the detected differences. Network and KEGG analyses revealed that the microbial community was very important for amino acid and carbohydrate metabolism and was slightly related to lignocellulose degradation during composting of the two substrate formulas. Bacillota, Pseudomonadota, and Bacteroidota affected multiple metabolic pathways during the composting process. The results expand the understanding of the microbial community and demonstrate that using MR for the cultivation of straw mushrooms is a low-cost and sustainable process.

## Data Availability

The original contributions presented in this study are included in the article/Supplementary Material. Further inquiries can be directed to the corresponding author. The sequence data are available on the NCBI website (access number: PRJNA1280709).

## Supplementary Materials

The following supporting information can be downloaded at https://www.mdpi.com/article/10.3390/microorganisms13102372/s1. Figure S1: Rarefaction curves of different treatments based on the number of operational taxonomic units (OTUs). Figure S2: Differentially abundant bacterial taxa identified by LEfSe. Linear discriminant analysis (LDA) effect size (LEfSe) was used to identify significantly enriched bacterial taxa among the different groups. (a) LDA score (log 10) bar plots demonstrating taxa with significant differences in relative abundance in the CW and MR formulas. Taxa with LDA threshold values > 3.0 are shown: CWP0 (green), CWPI (red), CWPII (blue), MRP0 (purple), MRPI (light blue), and MRPII (orange). (b) Cladogram representing the taxonomic relationships among the differentially abundant taxa identified by LEfSe. Taxonomic levels from phylum to genus are displayed, with colored nodes indicating significantly enriched bacterial groups in corresponding sample categories. Figure S3: Heatmap displaying the relative abundance of functional profiles at KEGG level 1. Table S1: Stages sampled during substrate preparation and *V. volvacea* cultivation.
