# Supplementary material for: Sustainable Recycling of Mushroom Residue as an Effective Substitute for Cotton Hull Waste in Volvariella volvacea Cultivation: Evidence from Physicochemical and Microbiome Analyses
Source: Microorganisms. 2025 Oct 15;13(10):2372. doi: 10.3390/microorganisms13102372 (PMC12566263; doi:10.3390/microorganisms13102372)
Supplement: Supplementary file 1 [file microorganisms-13-02372-s001.zip › microorganisms-3860509-supplementary.pdf]

Supporting Information For

Sustainable Recycling of Mushroom Residue: An Effective Substitute for Cotton Waste in *Volvariella volvacea* Cultivation Demonstrated by Physicochemical and Microbiome Analyses

Pattana Kakumyan [1,2,3†], Lin Yang[1†], Shunjie Liu[1], Kritsakorn Saninjuk[2,3], Qin Dong[1], Xueyu Pan[1], Changxia Yu[1], Yan Zhao[1*]

1 Institute of Edible Fungi, Shanghai Academy of Agricultural Sciences, Shanghai 201403, China; pattana.kak@mfu.ac.th (P.K.); ylin_jade@163.com (L.Y.); jasonliu86@foxmail.com (S.L.); maomao88719@163.com (Q. D.); panxueyu93@163.com (X. P.); ycx41529@163.com (C. Y.); jiandan289@126.com (Y. Z.)

2 School of Science, Mae Fah Luang University, Chiang Rai 57100, Thailand; kritsakorn.san@mfu.ac.th (K.S.)

3 Microbial Products and Innovations Research Group, Mae Fah Luang University, Chiang Rai 57100, Thailand

* Correspondence: jiandan289@126.com

This document contains:

One table: Table S1

Three figures: Figure S1, Figure S2, and Figure S3

Table S1. Stages sampled during substrate preparation and *V. volvacea* cultivation

Sampling stage code	Sampling time
BH, P0	Day 1, after prewetting
PI	Day 2, at the end of phase I composting
PII	Day 3, at the end of phase II composting
Fi	Day 8, mycelia covering the entire surface of the substrate
PF	Day 10, primordia formation stage
Ha	Day 14, first flush of mushroom harvesting

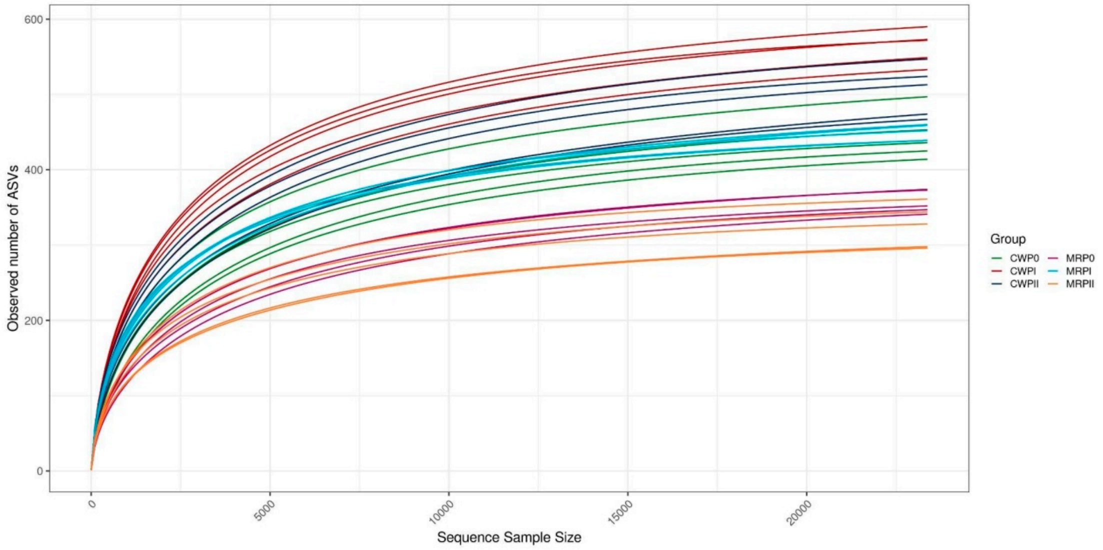

Figure S1. Rarefaction curves of different treatments on the basis of the number of operational taxonomic units (OTUs).

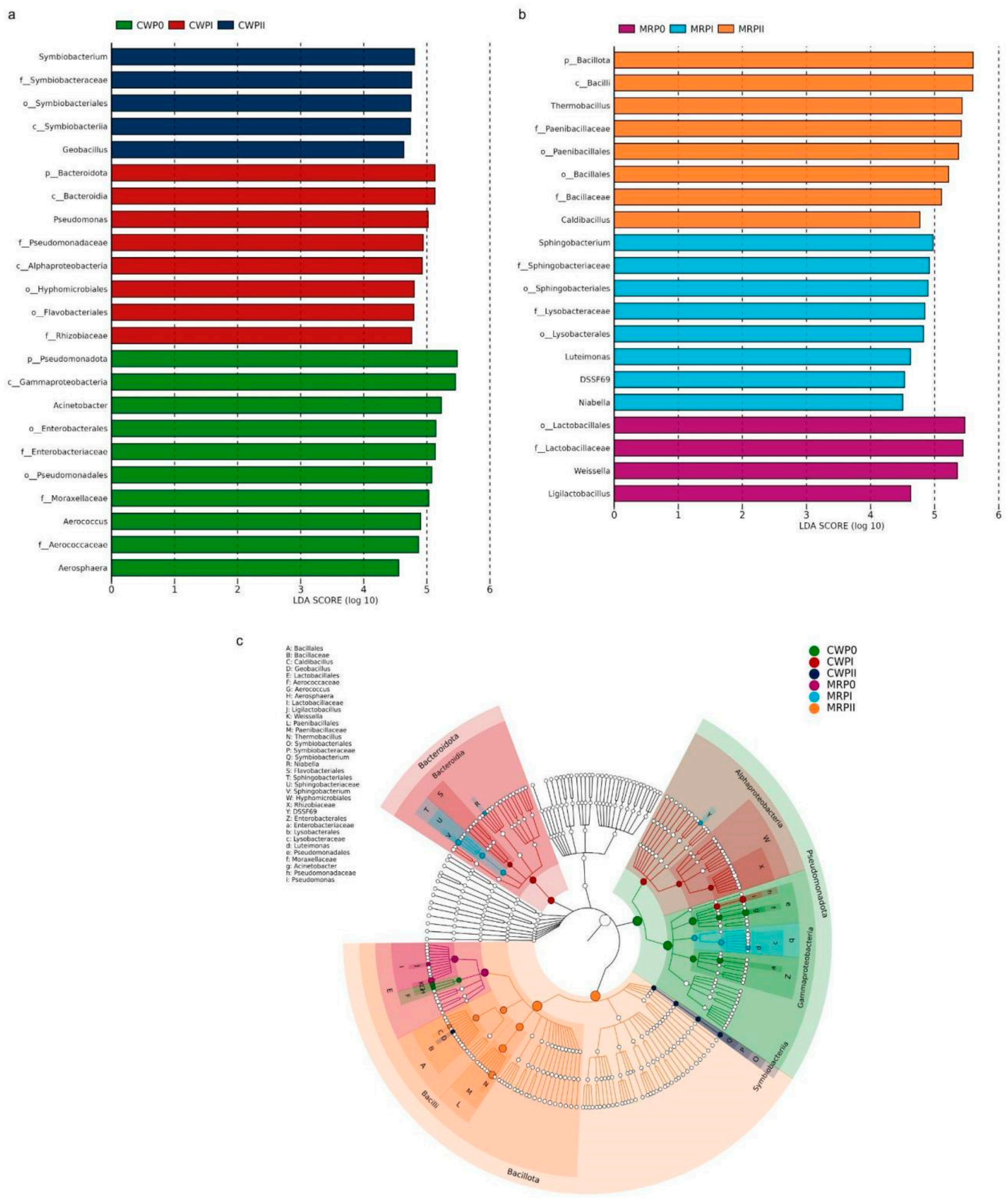

Figure S2. Differentially abundant bacterial taxa identified by LEfSe. Linear discriminant analysis (LDA) effect size (LEfSe) was used to identify significantly enriched bacterial taxa among the different groups. (a) LDA score (log 10) bar plots demonstrating taxa with significant differences in relative abundance in the CW and MR formulas. Taxa with LDA threshold values > 3.0 are shown; CWP0 (green), CWPI (red), CWPII (blue), MRP0 (purple), MRPI (light blue), and MRPII (orange). (b) Cladogram representing the taxonomic relationships among the differentially abundant taxa identified

by LEfSe. Taxonomic levels from phylum to genus are displayed, with colored nodes indicating significantly enriched bacterial groups in corresponding sample categories.

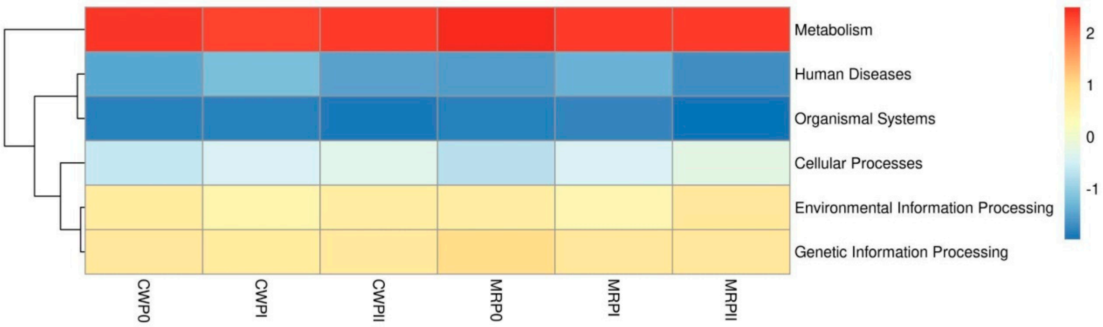

Figure S3. Heatmap displaying the relative abundance of functional profiles at KEGG level 1.
